# From plentiful to critically endangered: Demographic evidence of the artisanal fisheries impact on the smalltail shark (*Carcharhinus porosus*) from Northern Brazil

**DOI:** 10.1371/journal.pone.0236146

**Published:** 2020-08-06

**Authors:** Francisco Marcante Santana, Leonardo Manir Feitosa, Rosângela Paula Lessa

**Affiliations:** 1 Laboratório de Dinâmica de Populações Aquáticas (DAQUA), Universidade Federal Rural de Pernambuco (UFRPE), Unidade Acadêmica de Serra Talhada (UAST), Serra Talhada, PE, Brazil; 2 Laboratório de Dinâmica de Populações Marinhas (DIMAR), Universidade Federal Rural de Pernambuco (UFRPE), Departamento de Pesca e Aqüicultura (DEPAq), Recife, PE, Brazil; 3 Programa de Pós-Graduação em Biologia Animal, Universidade Federal de Pernambuco (UFPE), Recife, PE, Brazil; Consejo Nacional de Investigaciones Cientificas y Tecnicas (CONICET), ARGENTINA

## Abstract

The smalltail shark, *Carcharhinus porosus*, was the most abundant elasmobranch species in fisheries off Brazil’s northern coast (BNC) in the 1980s, but its population has been declining since the 1990s. For this reason, a demographic analysis is necessary to determine the extent of this decline and the fishing effect on the BNC’s population. Therefore, we performed a stochastic demographic analysis of the population in the BNC, and considered its global center of abundance. Smalltail shark specimens (*n* = 937) were collected with gillnets in Maranhão state, eastern BNC, in the 1980s with sizes ranging between 29.6 and 120.0 cm total length. Most of the individuals (90.6%) caught were juveniles (< 6 years-old), and the mortality and exploitation rates showed that the species was overexploited (92.3% above the fishing mortality corresponding to the population equilibrium threshold). The smalltail shark’s biological characteristics, such as slow growth and low fecundity, demonstrate that it is one of the least resilient species among similar sized coastal sharks in the region. All these factors yielded an annual decrease of 28% in the intrinsic population growth rate, resulting in a population decline of more than 90% in only 10 years, and much higher for the current period. This set of features comprising fishing recruitment occurring upon juveniles, overfishing, and intrinsically low resilience make the population unable to sustain fishing pressure and severely hamper biological recruitment, thus causing this drastic population decline. Furthermore, several local extinctions for this species in the northeastern and southeastern regions of Brazil highlight its concerning conservation scenario. Therefore, since similar fisheries characteristics occur throughout its distribution range, *C*. *porosus* fits the criteria E of the IUCN Red List for a critically endangered species and urgent conservation measures are needed to prevent its extinction in the near future.

## Introduction

Basic studies on elasmobranch populations are crucial for conservation status assessments and the establishment of management measures. These studies are particularly important for Data Deficient (DD) species for which little or no information is available, but populations might be under extinction risk [1,2]. An effective way to provide this information is to perform demographic analysis based on species-specific biological data. This has been done for several shark species to identify the major threats they face [3–5] employing stochastic demographic analysis and Monte Carlo simulations [3,6,7]. Consequently, this has enabled the incorporation of uncertainty and elasticity into population parameters, thus increasing the robustness of demographic analysis and enabling researchers to evaluate how populations would behave under different fishing scenarios.

However, demographic analyses are typically performed for highly fished species, especially those subjected to intense industrial fishing by developed countries’ fishing fleets [3,8]. Brazil, on the other hand, has an intense artisanal and semi-industrial fishery with elevated elasmobranch bycatch levels, and little to no regulation for almost ten years now [9]. In this context, only a handful of coastal shark species (*Isogomphodon oxyrhynchus* [4], *Carcharhinus signatus* [3]) were studied through demographic analyses and elasticities testing different exploitation scenarios. Nevertheless, 27 shark species are under some level of threat in Brazil even with population trends mostly unknown [10].

One of these is *Carcharhinus porosus* (Ranzani, 1839), a coastal shark that inhabits tropical waters, common in mud bottoms and near estuaries, not exceeding 150 cm total length (TL) [11]. It was considered to occur from the Gulf of Mexico to southern Brazil and from the Gulf of California to Peru [11,12]. However, a recent taxonomic review by Castro [13] resurrected its Pacific Ocean synonym, *C*. *cerdale*, as a valid species, thus restricting the distribution of *C*. *porosus* to the Western Atlantic Ocean. In fact, both are part of the *Carcharhinus dussumieri-sealei* group with similar sized sharks mentioned by Garrick [14] known to occur all over the world in coastal tropical waters and with recent taxonomic revisions [15–17]. Furthermore, *C*. *obsolerus*, a recently described species from this group is one of the few sharks already considered to be extinct [16], thus raising concerns regarding *C*. *porosus* conservation prospects in both short and long-term scenarios. In Brazil, *C*. *porosus* original area of distribution ranged from the northern coast to Cananeia beach in São Paulo, Southeastern Brazil [18–20]. Recent studies demonstrated that this occurrence pattern has changed dramatically with a significant decrease in the species’ distribution area throughout Brazil’s coast [21–23]. These declines caused by possible local extinctions restricted *C*. *porosus* occurrence to the northern coast of Brazil between Maranhão and Amapá states [21]. This area is considered its global center of abundance for the high proportion of individuals caught when compared to other regions where it occurs [24]. Recent data estimates show that the Amazon coast is likely the most important area for its conservation along its geographical distribution [23]. Notwithstanding, it was the dominant species of shark landed during the 1980s and 1990s in the Brazilian Northern coast (BNC), but it is now only the third most caught shark species in the region [22].

The BNC is considered one of the major fishing grounds for several highly exploited marine crustacean, teleost, and elasmobranch species, with two states in the region among the largest fish producers in Brazil. Pará state was the largest producer in 2011 with around 87,000 tons and Maranhão was the third with over 45,000 tons [25]. Three main types of fisheries exist in the area and cause major bycatch for *C*. *porosus* in different types of habitat. First, the artisanal gillnet fisheries targeting the Brazilian Spanish mackerel, *Scomberomorus brasiliensis*, and the acoupa weakfish, *Cynoscion acoupa*, which is characterized by a large artisanal fleet composed of wood boats with little fishing autonomy employing surface and midwater gillnets [20,26]. Second, the bottom trawl shrimp fishing operated mostly in an industrial scale targeting the pink shrimp *Farfantepenaeus subtilis* [27]. This fishery operates over long periods (40 to 90 days) with large metal boats (< 20 m long) with refrigerators [27,28]. Ships use single or double-rig otter trawl nets for five to seven hours employed between the 20 and 50 m isobaths within the continental platform outside the Amazon River mouth [27–29]. Third, industrial generalist fisheries targeting several teleost species, which uses similar settings to the bottom trawl shrimp fisheries, but employ gillnets ranging from 1 to 9 km in extension targeting large teleosts in general, especially the Laulao catfish *Brachyplatystoma vaillantii* [29,30].

Little information exists on the impacts these fisheries have had on the *C*. *porosus* population in the area, especially for the trawl and large teleost gillnet fisheries. During the 1980s, it corresponded up to 70% of the total catch weight and 43% of the number of individuals caught in the artisanal gillnet fisheries [19,20]. *Carcharhinus porosus* catches decreased from a CPUE of 2.87 kg/hour in 1990 to 0.43 kg/hour in the early 2000s [21,26]. Regarding the shrimp trawl fisheries, no catch data at the species level exists, but sharks were considered to be frequently caught and corresponding to 35.1% of the bycatch, including *C*. *porosus* [27,31]. The only data with identification at the species level points out that *C*. *porosus* corresponded to roughly 1.5% of the catch in the shrimp trawl fisheries prospections in the early 2000s [28].

Due to the great portion of juvenile individuals in the artisanal gillnet fisheries and the 85% biomass decrease observed in 2004, *C*. *porosus* was considered initially as threatened with extinction by the Normative Instruction 05/2004 [32]. In 2005, the species was considered overexploited, but not under extinction threat [33]. In 2014, the Brazilian government started to apply the International Union for the Conservation of Nature (IUCN) Red List Categories and Criteria to evaluate the national species, and their conservation statuses. Then, *C*. *porosus* was classified as critically endangered (CR) in Brazil due to the recent increase in fishing effort with the use of longer gillnets spanning over 10 km [34], its occurrence in several different large scale fisheries for shrimp and teleosts [30], and its decreased national occurrence area, and lack of population increase [21]. However, even though a few studies investigating *C*. *porosus* biological features such as age and growth [35], diet [36], reproduction [37,38], sexual dimorphism [39], distribution [23], and habitat use [40] exist, it remains DD according to the IUCN. Furthermore, genetic diversity data obtained from specimens in the Amazon coast point to a low allele diversity, which potentially indicates the effects of overfishing in the population [41]. Therefore, new data, especially considering the effects of fishing on its population, are paramount for *C*. *porosus* classification as a species of high risk of extinction and for the development of effective science-based conservation strategies.

To provide a population assessment for *C*. *porosus*, this study aims to estimate its demographic parameters in its global center of abundance. We also tested population responses to different fishing scenarios by calculating survival and fertility elasticities. Furthermore, we analyzed the *C*. *porosus* population’s recovery potential against other related species with similar total lengths by comparing published demographic parameters. We hypothesize that fishing recruitment on the second year of life, thus well before individuals reach sexual maturity and reproduce, is the most important cause for the observed decline of *C*. *porosus*. To demonstrate that, we expect to obtain negative values of population growth rate under the realistic fishing scenario, and positive population growth rate for the no fishing scenario. Finally, we applied the IUCN criteria to the data obtained for an accurate assessment of *C*. *porosus* conservation status.

## Materials and methods

### Ethics statement

Although we report data obtained with field research and animal sampling, no permit for this research was issued because no ethics committee existed at the time (early 1980s in Brazil) sampling took place. Furthermore, no field work was carried out nor animals were killed specifically for this study.

### Species biological information

**Fig 1:**
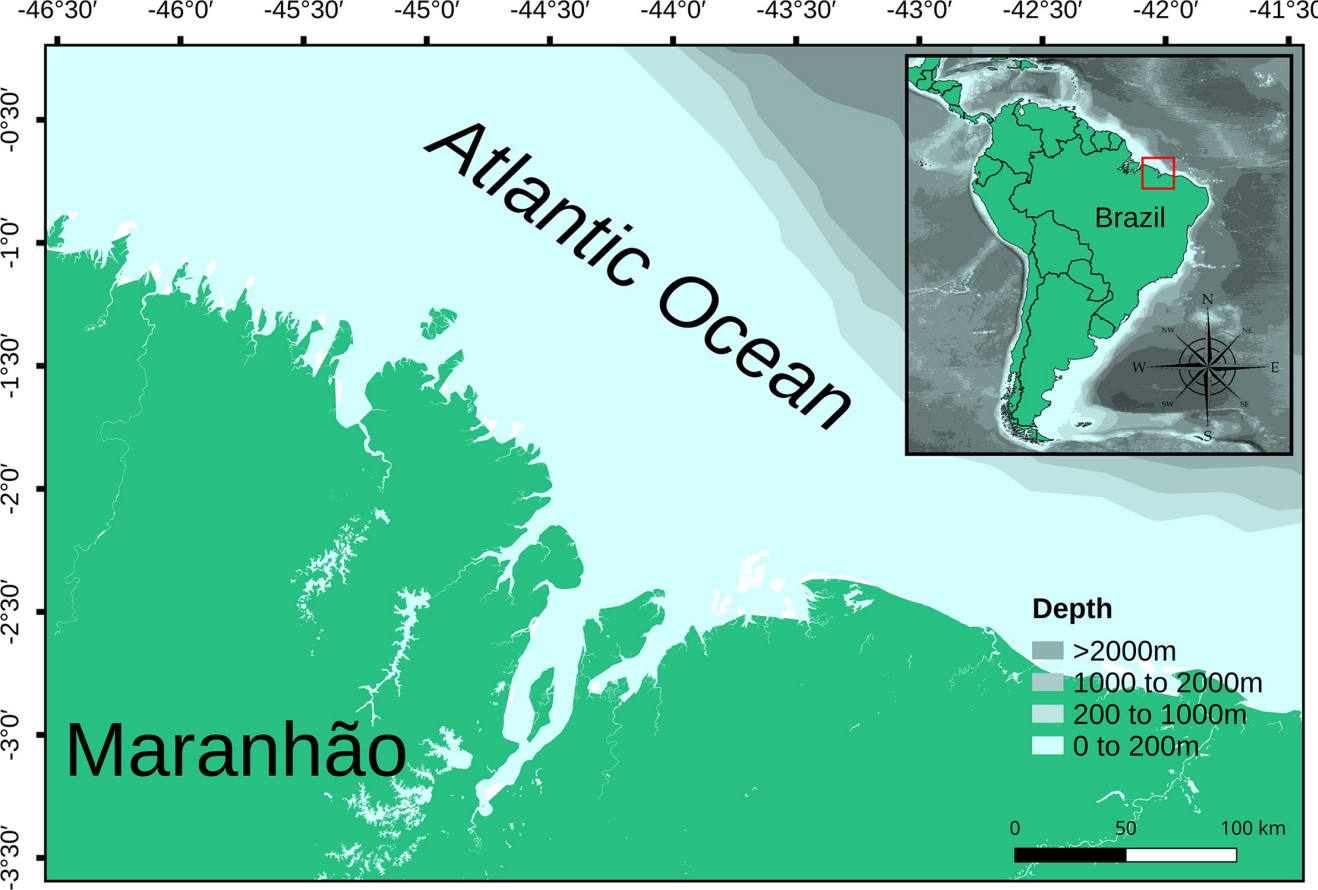
Location of *Carcharhinus porosus* catches in Maranhão state’s coast. Light blue areas correspond to the 200 m isobaths followed by the 1000 m, 2000 m and above 2000 m depth.

Data analyzed herein only corresponds to the written sample files from the experimental gillnet fisheries study carried out by Rosangela Lessa in the 1980s in Maranhão state. These data were collected with gear on the same settings as the one employed in the artisanal fishery targeting *S*. *brasiliensis* and *C*. *acoupa* in Maranhão state’s coast. *C*. *porosus* specimens analyzed were caught with 900 m long and 7.5 m high gillnets, with 8.0 cm stretched mesh between June 1984 and November 1987 in the BNC (46ºW to 43º40’00”W) (Fig 1). Sex and TL (cm) were registered for each individual and the biological data were retrieved from published literature [20,35,37,38]. Since there are no data on the specimens captured by the shrimp trawl fisheries, all conclusions drawn in this study are specifically applied to the artisanal gillnet fishery.

*Carcharhinus porosus* reproductive biology in Maranhão state is well known [26,37,38], with males and females reaching sexual maturity at 70 cm and 71 cm total length, respectively. Furthermore, the embryo sex ratio is 1:1, fecundity varies from 1 to 10 embryos (average = 5.94 ± 2.26) with a biennial reproductive cycle. These values yielded an average fecundity of 1.48 (SD = ±0.57) female embryos per pregnant individual per year. We used a normal distribution with this mean and standard deviation as uncertainties for the stochastic demographic analysis.

Growth parameters and population structure were estimated for *C*. *porosus* in Maranhão by Lessa & Santana [35]. Their results showed no significant age and growth differences between sexes, thus yielding the following von Bertalanffy growth function (VBGF) growth parameters: *L*_*∞*_ = 136.4 cm TL; *k* = 0.077 yr^-1^, and *t*_*0*_ = -3.27 years. The maximum observed age (*t*_*max*_) was of 12 years, with sexual maturity (*t*_*mat*_ converting the length at maturity of 71 cm for females in age through the inverted VBGF equation) occurring at 6 years of age. We used discrete probability distributions of both ages as uncertainties in the stochastic analysis, with probability (*p*) of 0.50 for the ages *t*_*max*_ and *t*_*mat*_, and *p* = 0.25 for a year prior to *t*_*mat*_ or *t*_*max*_, and other *p* = 0.25 for a year after *t*_*mat*_ or *t*_*max*_.

### Demographic analysis

Natural mortality rates (*M*) were estimated by nine age-independent and two age-dependent methods, based on several life cycle parameters for the species (Table 1). Furthermore, total mortality rates (*Z*) were calculated by catch curves (one using total lengths converted to age by the inverted VBGF and other based on the sample age structure), and by the methods of Beverton & Holt [42] by length and age. The stochastic analysis was estimated as uncertainties from both the eleven values of *M* and the four values of *Z* obtained, with discrete probability distributions of 0.0909 for each *M* value and 0.250 for each *Z* value. Although we understand the limitations of demographic analysis to estimate extinction risk due to density-dependent factors on population dynamics [43], we use it as a tool to unravel the effects of fisheries in the *C*. *porosus* population at the BNC.

**Table 1:** Methods and formulae used to estimate natural mortality (*M*) of *Carcharhinus porosus* in Northern Brazil.

Method	Equation
Pauly [44]	$\ln M=-0.0152-\left[0.279\;\ln\left(L_{\infty }\right)\right]+\left[0.6543\;\ln\left(k\right)\right]+\left[0.463\;\ln\left(Temp\right)\right]$
Jensen [45]	*M* = 1.6*k*
Jensen [45] 2	*M* = 1.5*k*
Jensen [45] 3	$M=\frac{1.65}{t_{mat}}$
Rikhter & Efanov [46]	$M=\left(\frac{1.521}{t_{mat}{}^{0.72}}\right)\left(-0.155\right)$
Hoenig [47] for Teleosts	ln *M* = 1.46−1.01[ln(*t*_max_)]
Hoenig [47] for Cetaceans	ln *M* = 0.941−0.873[ln(*t*_max_)]
Hewitt & Hoenig [48]	$M=\frac{4.22}{t_{\max}}$
Mollet & Cailliet [49]	$M=\frac{\ln\left(0.01\right)}{t_{\max}}$
Peterson & Wroblewski [50]	*M* = 1.92*W_t_*^−0.25^
Chen & Watanabe [51]	$M_{\left(t\right)}=\frac{k}{\left[1-e^{-k\left(t-t_{0}\right)}\right]}$ for ages up to 1—*t*_*mat*_ (6 years), and $M_{\left(t,t_{\max}\right)}=\left[\frac{1}{\left(t_{\max}-t\right)}\right]\times \ln\left[\frac{\left(e^{k}t_{\max}-e^{k}t_{0}\right)}{\left(e^{k}t-e^{k}t_{0}\right)}\right]$ for *t* ≥ 7 years

*L*_*∞*_ - asymptotic length, *k*–growth coefficient, *Temp*—mean annual water temperature (28°C), *t*_*mat*_−age of female maturity, *t*_*max*_−maximal age observed, and *t*_*0*_ –theoretical age at zero length.

We averaged total (*Z*) and natural mortality (*M*) estimates solely to calculate fishing mortality (*F*), which is the difference between the former and the latter. On the other hand, we calculated survival (*S*) for each of the estimated total mortalities from the equation described by Ricker [52]:

$$S=e^{-Z}$$

In addition, we calculated the exploitation rate (*E*), which enables the observation of over (when > 0.5) or under (< 0.5) exploitation, with the equation: *E* = *F/Z* [44]. All calculations were done in Microsoft Excel.

We employed the age-based Leslie matrix (*L*) from the PopTools program [53] in Microsoft Excel to calculate population elasticities. *L* was a Leslie population projection matrix, adopting a pre-breeding census (reproduction first, then survival):

$$L=\left[\begin{array}{ccccc} f_{0} & f_{1} & f_{2} & \ldots & f_{x}\\ s_{0} & 0 & 0 & 0 & 0\\ 0 & s_{1} & 0 & 0 & 0\\ 0 & 0 & \ldots & 0 & 0\\ 0 & 0 & 0 & s_{x-1} & 0 \end{array}\right]$$

In which *f_x_ = s_x_×m_x_* and *s_x_* are the annual survivorship term for age *x*, and *f_x_* represents age-specific fecundity rate per capita. This method employs matrix algebra to calculate *λ* values for each age in the population [54]. Furthermore, this method enables us to calculate elasticities, which are defined as the proportional sensitivities of the population to a given matrix element (i. e. fishing recruitment) [55].

From this Leslie matrix, we calculated the population parameter values of *R*_*0*_ (expected number of replacements or net reproductive rate), *T* (generation time or time for increase in *R*_*0*_), *r* (intrinsic rate of population growth or rate of increase), and *λ* (finite rate of population growth) [54,56]. Furthermore, Monte Carlo simulations were used to estimate these parameters and the used elasticities (*e_ij_*) corresponding to the survivorship by age and fertility. For elasticity estimates of *λ* (proportional change in *λ* for proportional changes in matrix *L*, denominated *a*_*ij*_), the values of each age and fertility are additive. Therefore, the sum of these elasticities defines the proportional contribution of *a*_*ij*_ to the overall population *λ*. Elasticity was calculated as:

$$e_{ij}=\frac{a_{ij}}{\lambda }\frac{\partial \lambda }{\partial a_{ij}}$$

From these results, we created three scenarios to estimate demographic parameters. First, a no-fishing hypothesis with a constant *M* value was used for the age classes. In the second, the closest to reality, the *F* value was included through the fishing recruitment age (highest number of individuals from the same age caught by the fishery). Third, we analyzed the influence of juvenile captures on the species’ demography with a hypothetical scenario, in which fisheries only catch adult individuals. Therefore, fishing mortality rates are used only for age classes above six years.

For scenarios with negative *r* values, we estimated the number of survivors for age *t* (*N*_*t*_), corresponding to 10 years in the population (*N*_*10*_) and three times the generation time (*N*_*3T*_), considering an initial survival (*N*_*0*_) of 1 through the equation described by Otway et al. [57]: $N_{t}=N_{0}e^{rt}$. This calculation enables us to estimate the population decline (*D*_*t*_ *= 1-e*^*rt*^) according to the scenarios employed and their comparison with criterion E of the IUCN conservation status assessment protocol: Quantitative analysis showing the probability of extinction in the wild is at least 50% in three generations [58].

In addition, the intrinsic rebound potential of productivity (*r*_*Z*_) was estimated according to Smith et al. [59], and the fishing mortality rate necessary to drive the species to extinction (*F*_*extinct*_) was calculated according to Garcia et al. [60]. This mortality rate is equivalent to the maximum intrinsic rate of population increase (*r*_*max*_), which is a standard measure of population productivity and of extinction risk [1]. All these calculations were performed in Microsoft Excel.

## Results

**Fig 2:**
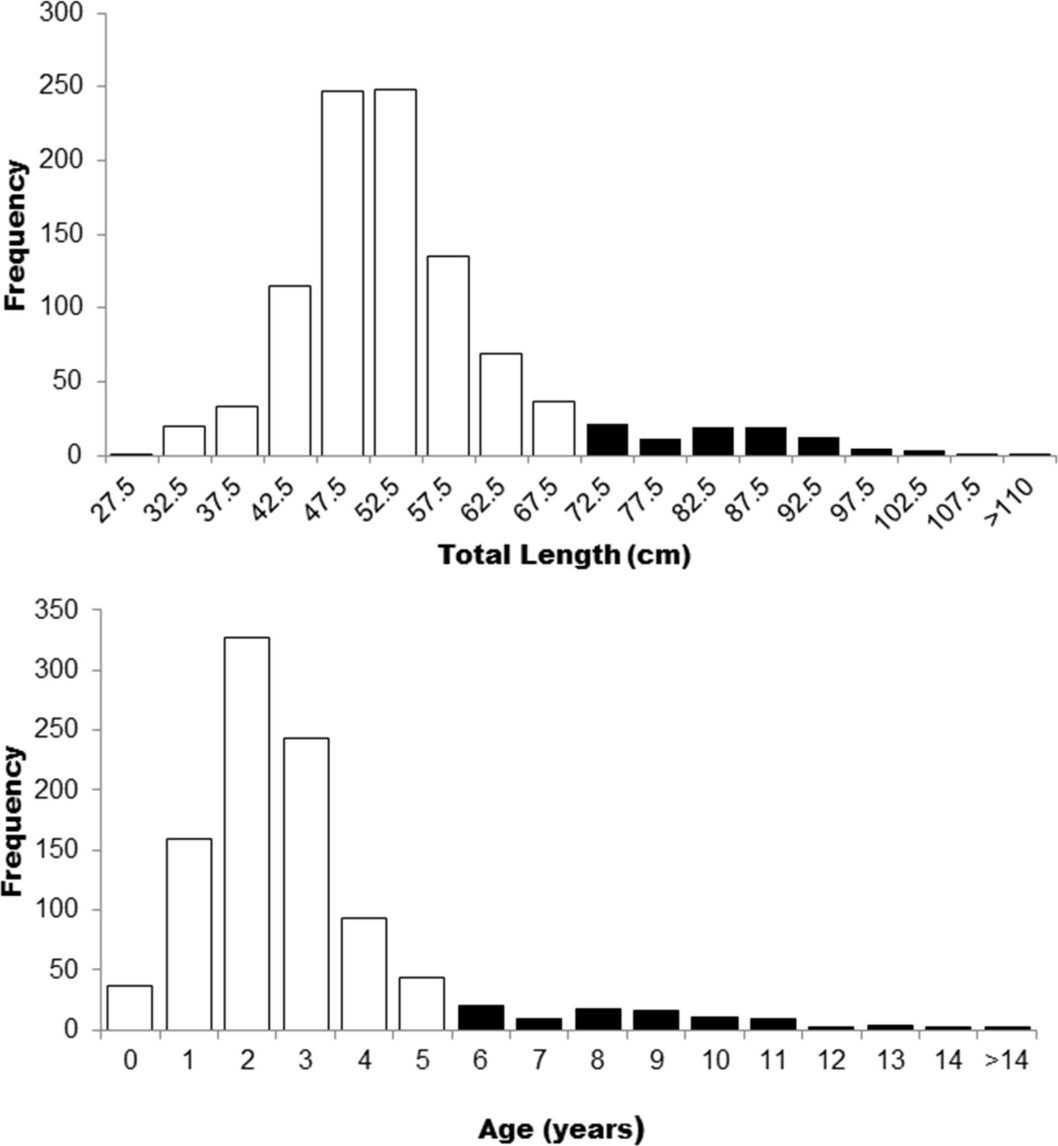
Total length (a) and age (b) frequency distributions of juvenile and adult individuals of *Carcharhinus porosus* from Northern Brazil. White columns correspond to juveniles and black columns to adults.

We analyzed catch and biological information for 937 individuals of *Carcharhinus porosus*. Since no significant differences exist in the frequency distributions between sexes by length and age [37], both sexes were analyzed together. Total lengths varied from 29.6 to 120.0 cm TL, with a larger frequency between 45 and 55 cm (Fig 2A). Ages estimated according to the inverted VBGF varied between 0 and >14 years with the mode indicating fishing recruitment at 2 years-old with a juvenile frequency (< 6 years) of 90.6% (Fig 2B).

The smaller value estimated for *M* was established by the Jensen [45] 2 method and the greatest by the Mollet & Cailliet [49] method, resulting in an average of 0.261 (*S* = 0.770) from the eleven methods (Table 2). Total average mortality rate (*Z*) was 0.656 (*S* = 0.519) and, when subtracted by *M*, yielded a fishing mortality *F* = 0.395 and an exploitation rate *E* = 0.602, thus indicating overexploitation.

**Table 2:** *Carcharhinus porosus* natural and total mortalities, as well as survival rates estimated by several methods.

Method	Mortality	Survival
Natural (*M*)		
Pauly	0.222	0.801
Rikhter & Efanov	0.250	0.779
Hewitt & Hoenig	0.301	0.740
Hoenig–teleosts	0.300	0.741
Hoenig–cetaceans	0.256	0.774
Jensen 1	0.123	0.884
Jensen 2	0.116	0.891
Jensen 3	0.263	0.769
Mollet & Cailliet	0.329	0.720
Peterson & Wroblewski	0.540–0.247	0.583–0.781
Chen & Watanabe	0.346–0.062	0.708–0.940
Total (*Z*)		
Catch curve by length	0.677	0.508
Catch curve by age	0.749	0.473
Beverton & Holt by length	0.519	0.595
Beverton & Holt by age	0.681	0.506

The mortality rate corresponding to population equilibrium (*Z*’), which is the constant total mortality *Z* that would be enough to keep the population under sustainable levels (corresponding to a value of *r* = 0 and *λ* = 1), was estimated at 0.271 (*S* = 0.763). Considering *M* = 0.239, the equilibrium fishing mortality rate (*F’*) would be equal to 0.032. When compared to the estimated *F* value (0.417), we obtain an overexploitation degree of 92.3%.

**Fig 3:**
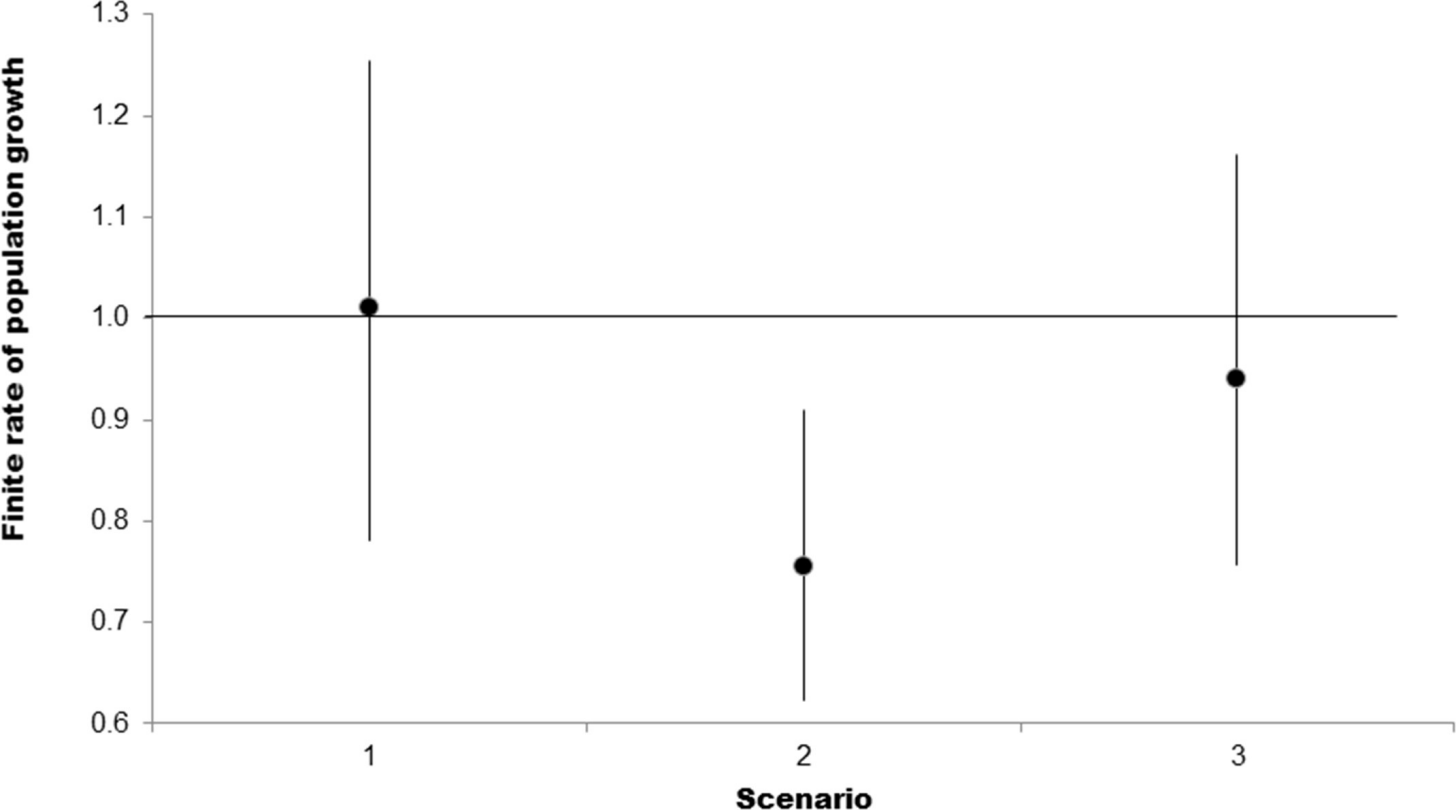
Finite rate of population growth (*λ*) mean (points) and 95% confidence intervals (bars) for different scenarios of *C*. *porosus*. Dashed line corresponds to the population equilibrium (*λ* = 1).

**Fig 4:**
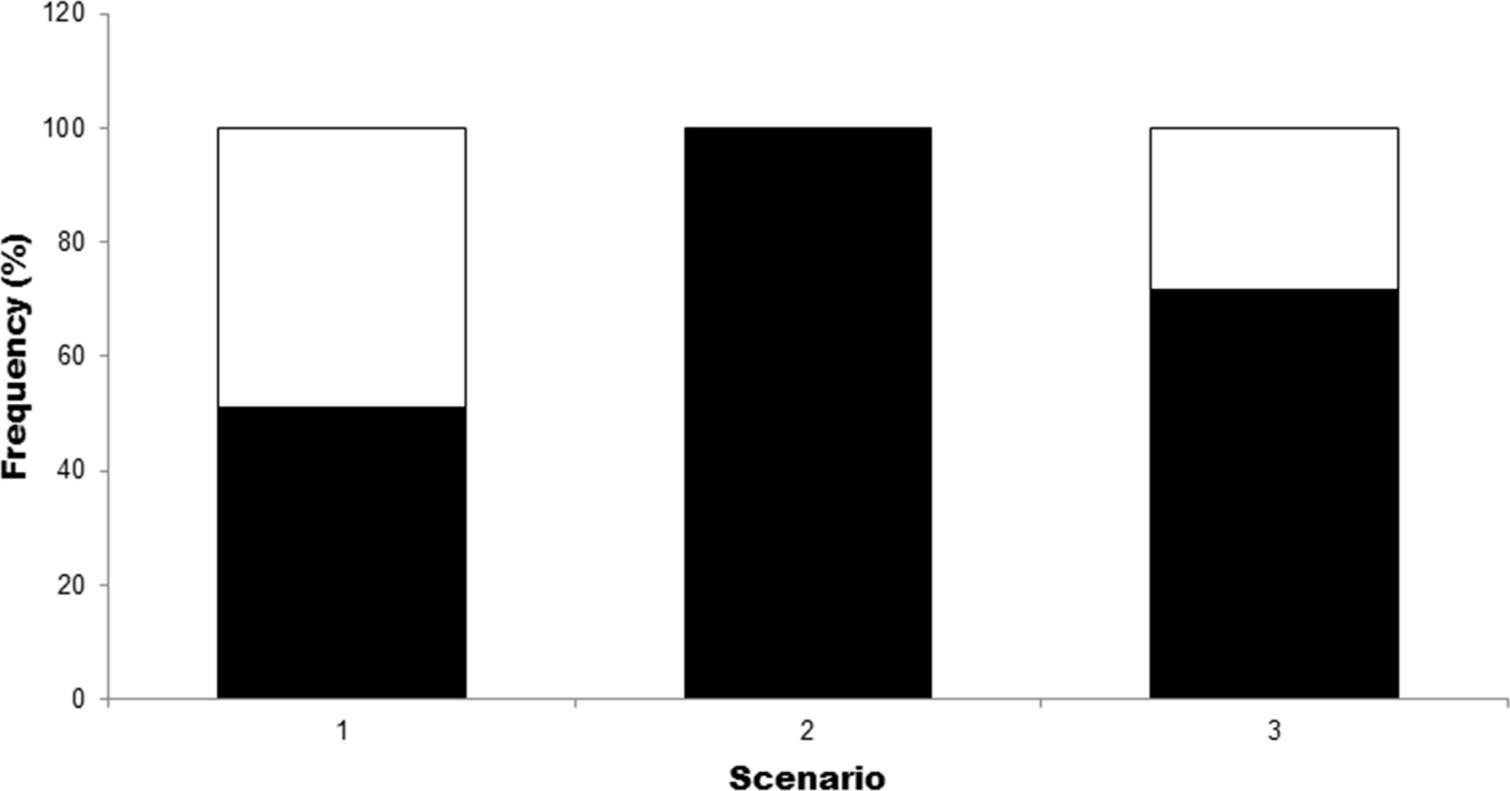
Proportions of finite rate of population growth (*λ*). Smaller values (black) and larger (white) than 1 of the 1000 simulations by scenario for *Carcharhinus porosus*.

In the first scenario of the elasticity analysis (no fishing exploitation), *C*. *porosus* population would be close to equilibrium (*λ* = 1, *r* = 0), with a slight annual increase of 0.3%, thus indicating the intrinsic natural vulnerability of the population. The greatest elasticity corresponded to juvenile survival (*e*_*2*_), which demonstrates the importance of this life stage for the population demography (Table 3). The 1,000 simulations performed in this scenario reveal a similarity in the proportions of the *λ* values, with 51.1% of it indicating population increase (*λ* > 1) and a decrease for the rest (*λ* < 1) (Figs 3 and 4).

**Table 3:** Demographic analysis for *C*. *porosus* with extrapolations for the next three generations and 10 years.

Scenario	*λ*	*r* (year^-1^)	*R*_*0*_	*T* (years)	*e*_*1*_	*e*_*2*_	*e*_*3*_	*3T* (years)	*N*_*3T*_	*D*_*3T*_	*N*_*10*_	*D*_*10*_
1	1.011 (0.780/1.254)	0.003 (-0.248/0.226)	1.588 (0.123/5.720)	8.030 (6.750/9.264)	0.126	0.628	0.246	24.090	-	-	-	-
2	0.756 (0.624/0.910)	-0.285 (-0.472/0.095)	0.163 (0.022/0.525)	7.737 (6.537/8.891)	0.131	0.654	0.215	23.211	0.001	0.999	0.058	0.942
3	0.940 (0.756/1.161)	-0.068 (-0.279/0.149)	0.809 (0.111/2.578)	7.207 (6.004/8.601)	0.140	0.701	0.158	21.620	0.229	0.771	0.505	0.495

Demographic parameters (*λ*, *r*, *R*_*0*_, and *T*) with respective 95% confidence intervals between parenthesis (lower/upper), elasticities (*e*_*1*_ = sum of elasticities of fertility, *e*_*2*_ = sum of juvenile survival and *e*_*3*_ = sum of adult survival), three times the generation time (*3T*); proportion of survivals at the age corresponding to three generations (*N*_*3T*_) and 10 years (*N*_*10*_), and respective declines (*D*_*3T*_ and *D*_*10*_) by scenario 1 (constant *M* for age classes), 2 (*Z* from the fishing recruitment age at two years old), and 3 (*Z* from the hypothetical fishing recruitment age equal to the age at maturity of six years old).

When the fishing exploitation is evaluated from the age of fishing recruitment (2 years) (Scenario 2), the inclusion of *F* yields a population decrease of around 28% per year with all simulations resulting in *λ* values smaller than 1. Therefore, in these conditions, there is no perspective of population increase nor equilibrium (Table 3; Figs 3 and 4). In addition, this scenario evidences that the catch of juvenile individuals and the high fishing mortality rate starting at 2 years old caused a major population decline in *C*. *porosus*. The estimated value of *r* in this scenario reveals that only 5.8% of the population survives for the first 10 years of life and, in three generations, there are only 0.1% of the initial cohort left, resulting in population declines of almost 100% during these periods (Table 3).

Considering the hypothesis that fishing recruitment started at the age of maturity (6 years) (Scenario 3), the population would continue to decline, but at a much lower rate (approximately 6.8% per year) (Table 3). The population decline at this scenario would be much smaller than in scenario 2, with a decline of 49.5% for the first 10 years, and 77.1% for three generations (Table 3). Of the 1,000 simulations performed for *λ*, 71.6% reveal population declines (Figs 3 and 4), thus indicating that the fishing mortality in scenario 2 is so high that, even capturing only adult individuals, the population would still decline.

**Fig 5:**
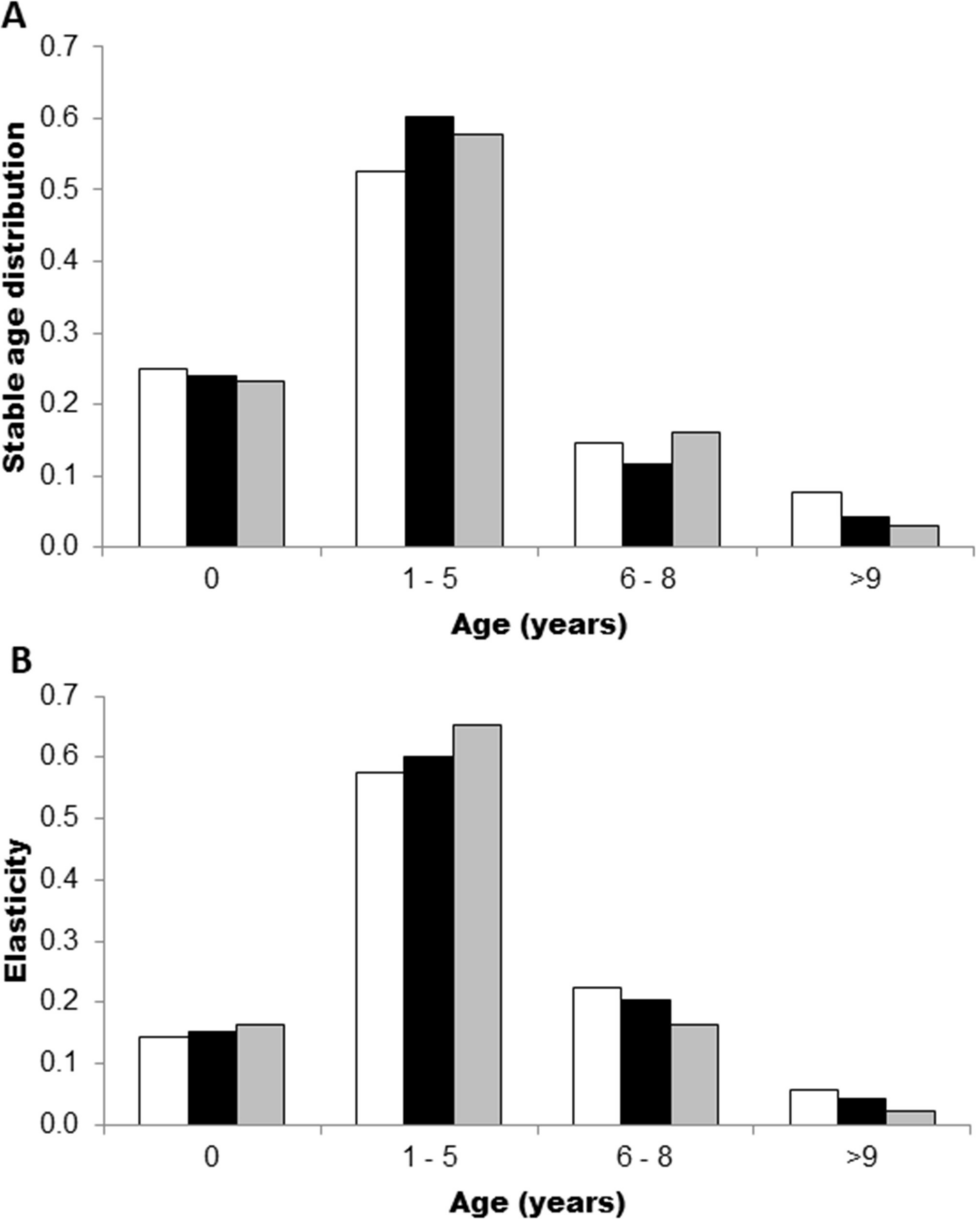
Stable age distribution (a) and elasticity (b) by stage of life cycle of *C*. *porosus*. Scenarios are represented by gray (1), white (2) and black (3) columns. Life stages are separated in neonates: 0; juveniles: 1–5 years; adults: 6–8 years, and large adults: >9 years).

For the three scenarios, the most important age classes for *C*. *porosus* demography were between 1 and 5 years. These classes represent more than 50% of the stable age distribution and survival elasticities (Fig 5), since survival of these individuals is fundamental for the reproductive stock. Furthermore, the intrinsic rebound (*r*_*z*_) estimated for *C*. *porosus* was of 0.048, and the *F*_*extinct*_ value of 0.254, which is inferior to the estimated *F* value, thus indicating the species overexploitation.

## Discussion

### Demographic inferences

Based on the demographic analysis, *C*. *porosus* decline in the BNC was caused by intense overfishing. All the information obtained by this study points toward a sharp decline in *C*. *porosus* population in its global center of abundance and the most important region for this species conservation in the world [23,24]. In the BNC, gillnet fisheries in the 1980s caught essentially juvenile *C*. *porosus*, especially those with 2 years old, who accounted for 37.8% in these fisheries (Fig 2B). Catching individuals 4 years before they reach sexual maturity causes a significant decline in the reproductive stock, also reducing biological recruitment levels in the population, which would help to maintain its sustainability. This pattern can be found in the same area for similar-sized species such as *I*. *oxyrhynchus* [4], causing significant declines in their populations.

In the smalltail shark’s case, we estimated the fishing mortality rate for this species to be almost 100% higher than the population could withstand. Furthermore, we obtained an exploitation rate greater than sustainability (*E* > 0.5), which is also corroborated by the *F* value above *F*_*extinct*_. Therefore, fisheries overexploitation magnified the population decline already caused by the fishing recruitment of juveniles. Indeed, several demographic studies with sharks reveal overfishing as one of the main causes for population declines [3,4,7,59]. This scenario is worsened if we consider the existence of the shrimp and teleost trawl fisheries, which have not been analyzed here but likely cause severe negative impacts on the *C*. *porosus* population as well [27,28,30].

In addition to overfishing and the high incidence of juveniles in the catches, *C*. *porosus* is an intrinsically low resilient species and highly vulnerable to fishing exploitation, mainly due to its biological characteristics. Even though Branstetter [61] classified *C*. *porosus* as a species with small size (*L*_*max*_ ~ 100 cm TL) and fast growth (> 30% of birth length in the first year of life), its life history traits are actually the opposite. *Carcharhinus porosus* has one of the longest juvenile phases when compared to several species of small coastal sharks, thus taking longer to reach sexual maturity. Indeed, when we added its high longevity and slow growth to its low fecundity, we obtained a small productivity value (*r*_*z*_ = 0.051) (Table 4). Furthermore, *C*. *porosus* productivity was closer to that of large coastal sharks (*r*_*z*_ < 0.04), which are generally low [59]. In fact, its *r*_*z*_ value is much inferior to the one expected for similar-sized coastal sharks (Table 4).

**Table 4:** Life history characteristics estimated for coastal shark species by different authors.

Species	*TL*_*max*_	*k*	*t*_*max*_	*t*_*mat*_	*m*_*x*_	*r*_*Z*_	References
*Isogomphodon oxyrhynchus*	141.0	0.121	12	6.6	1.21	0.053	Lessa et al. [62,63]
*Carcharhinus acronotus*	132.0	0.120	16	7.1	1.00	0.053	Hazin et al. [64]; Barreto et al. [65]
*Carcharhinus porosus*	120.5	0.077	12	6.1	1.48	0.051	Lessa & Santana [35]; Lessa et al. [37]
*Rhizoprionodon terranovae*	115.0	0.359	10	4.2	1.33	0.080	Cortés [56]
*Rhizoprionodon porosus*	110.0	0.171	10	3.3	2.50	0.098	Machado et al. [66]; Lessa et al. [67]
*Rhizoprionodon lalandii*	76.5	0.301	6	2.6	1.20	0.108	Lessa et al. [67]; Lessa [68]
*Sphyrna tiburo*	103.0	0.340	7	2.2	4.50	0.130	Parsons [69]
*Carcharhinus leucas*	285.0	0.076	27	15	3.25	0.027	Smith et al [59]; Branstetter & Stiles [70]; Pirog et al [71]
*Negaprion brevirostris*	320.0	0.057	25	12.7	4.25	0.034	Smith et al [59]; Feldheim et al [72]
*Galeocerdo cuvier*	430	0.202	28	11	5.6	0.043	Smith et al [59]; Winter & Dudley [73]; Whitney & Crow [74]

Maximum total length in cm–*TL*_*max*_, von Bertalanffy growth coefficient by year–*k*, Age at maturity—*t*_*mat*_ and maximum observed—*t*_*max*_ in years, average fertility of pregnant females from female embryos per year–*m*_*x*_, and intrinsic rebound potential of productivity (*r*_*Z*_). All data regard females.

Smith et al. [59] estimated the yield for 26 Pacific shark species and determined that small coastal sharks tend to have *r*_*Z*_ values higher than 0.08, denoting their high resilience. Indeed, Cortés [75] and Brewster-Geisz & Miller [76] describe the strong relationship between the vulnerability and the survival of juveniles, age at maturity, and other life history traits in the population parameters of shark species. Furthermore, Liu et al. [77] and Branstetter [61] argue that sharks with an earlier sexual maturity tend to maintain the population in equilibrium more easily even with a somewhat high fishing effort. This is the case because specimens are usually fished after the first reproduction, thus ensuring their contribution to the population. Therefore, there is a direct relationship between the duration of the juvenile phase and the vulnerability of a shark species.

As demonstrated by the mortalities and exploitation rate results, the BNC’s *C*. *porosus* population was already heavily overfished in the 1980s. Furthermore, the higher elasticity values for all scenarios tested point that the juvenile phase (1 to 5 years of age) is the most important stage for this species maintenance and individuals between these ages represent 86.9% of the population in these fisheries. Further analyzing the species’ resilience to fisheries, we calculated its intrinsic rebound potential (*r*_*z*_ = 0.051) and generation time (*T* = 7.9 years). Both values are similar to the ones found by Cortés [75] for *C*. *porosus* and, when compared to other similar-sized species, demonstrate how susceptible it is to fisheries exploitation. In fact, species already globally endangered such as *Sphyrna lewini* and *C*. *longimanus*, have smaller generation times than *C*. *porosus*, thus reproducing faster. On the other hand, *r*_*z*_ was a little higher than the one obtained for *I*. *oxyrhrynchus* (*r*_*z*_ = 0.039) [4], but still confirming its low resilience to fisheries.

Unlike other small to medium-sized coastal shark species (i.e. *Rhizoprionodon* spp.), *C*. *porosus* follows the same trends regarding life history features present in other *Carcharhinus* species such as *C*. *acronotus* and *C*. *brevipinna* [75], and the closely related *I*. *oxyrhynchus* [4]. When compared to other similar-sized species, the population parameters of *C*. *porosus* are concerning (Table 5). For example, its estimated maximum size is the smallest, but its generation time is higher and its survival rates are smaller than the ones of larger species such as *C*. *acronotus*, *C*. *sorrah*, and *C*. *tilstoni*. In addition, when the elasticity results are compared, the dependence on the juvenile life stage is similar to large coastal species such as *C*. *leucas* and *Galeocerdo cuvier*. Therefore, *C*. *porosus* is one of the most naturally at-risk species of *Carcharhinus* in the world, and the fishing pressure has deteriorated its population.

**Table 5:** Comparative finite rate of population increase (*λ*), generation time (*T*, in years) and elasticities (*e*_*1*_ = sum of elasticities of fertility, *e*_*2*_ = sum of juvenile survival and *e*_*3*_ = sum of adult survival) for coastal Carcharhinidae sharks used only natural mortality.

Species	*λ*	*T*	*e*_*1*_	*e*_*2*_	*e*_*3*_
*Carcharhinus acronotus*	0.847	4.2	0.191	0.472	0.336
*Carcharhinus amblyrhynchus*	0.941	9.6	0.094	0.609	0.297
*Isogomphodon oxyrhynchus*	0.950	9.2	0.110	0.662	0.227
*Carcharhinus limbatus*	0.974	10.0	0.093	0.600	0.306
*Carcharhinus leucas*	0.998	21.6	0.044	0.774	0.181
Carcharhinus porosus*	1.013	8.0	0.126	0.628	0.246
*Carcharhinus plumbeus*	1.022	19.8	0.048	0.693	0.259
*Carcharhinus obscurus*	1.030	26.2	0.037	0.679	0.285
*Carcharhinus brevipinna*	1.037	10.4	0.088	0.614	0.298
*Rhizoprionodon terranovae*	1.056	4.9	0.169	0.475	0.356
*Negaprion brevirostris*	1.064	16.4	0.057	0.700	0.242
*Rhizoprionodon taylori*	1.073	2.9	0.261	0.227	0.512
*Carcharhinus porosus*	1.086	8.4	0.107	0.582	0.312
*Carcharhinus sorrah*	1.093	4.3	0.194	0.370	0.435
*Carcharhinus tilstoni*	1.145	6.0	0.145	0.419	0.436
*Galeocerdo cuvier*	1.246	10.9	0.084	0.699	0.217

All values were obtained from Cortés [75], except for *Isogomphodon oxyrhynchus* [4] and *Carcharhinus porosus* in the present study (*).

### Conservation problems and future strategies

Our results are strong evidence for the deleterious effect of fishing on *C*. *porosus* BNC population, thus being responsible for its decline. Furthermore, recent studies demonstrate that the shark community in the BNC seems to have also suffered significant changes. The once most abundant species *I*. *oxyrhynchus* and *C*. *porosus* are now severely depleted [78], and were substituted by others with a higher resilience to fisheries such as *R*. *porosus* and *C*. *acronotus* [22]. Since fishing recruitment occurred at two years old (Fig 2B), the population could not sustain this fishing pressure and biological recruitment was severely hampered, thus evidencing its sharp population decline. In addition, the perspective worsens with the estimates of local extirpations in the Southeast and Northeastern regions of Brazil where catches have not been reported for decades [21].

Even though legislation has tried to follow along the conservation needs to manage this species’ populations by prohibiting its catches since 2004, no effective actions have been taken so far. In fact, the management plans that should have been developed when the species was listed in Annex I of the Normative Instruction No. 5 from 2004 [32] were never implemented and no population recovery has been observed so far. This led *C*. *porosus* to be considered as critically endangered in the current Brazilian Red List of endangered fishes and aquatic invertebrates (Ordinance 445/2014) [21,79]. Therefore, it is clear that the necessary information to apply management strategies already existed since the early 2000s, but the overall inaction of the environmental agencies and the Brazilian government were key to the current state of *C*. *porosus* populations in Brazil.

Despite the existence of catch and trade prohibitions, recent studies have demonstrated that several highly endangered species, including *C*. *porosus*, are continually caught in the BNC [22,80,81]. Furthermore, fishers report the lack of dialogue between the environmental agencies and their communities, as well as the virtually complete absence of inspections in the largest fishing ports of the region [78]. Since the fisheries in the area are mainly artisanal with traditional communities being an important factor in this equation, fishing ports are scattered throughout the area, thus making inspections and the reporting of endangered species extremely difficult. Furthermore, the lack of data on the semi-industrial and industrial shrimp and general teleost trawl fisheries that operate in the continental platform of the Amazon coast are extremely concerning and a key aspect that needs to be addressed by the responsible authorities.

Overall, fisheries management in Brazil is extremely flawed with the statistics program being discontinued since 2011, and no reliable information for any type of fishing activity exists ever since [9]. The only updated information on fishing activities in the area come from Mourão et al. [34] and Almeida et al. [82], who identified an average three-fold increase in the length of gillnets used in the BNC to overcome the decreased productivity of the target species [83]. Gillnets targeting *S*. *brasiliensis* now range from 3 to 9 km long [34], while the ones used in the *C*. *acoupa* and *Sciades parkeri* fisheries range from 3 to 15 km long [82]. Furthermore, artisanal fishers operate in vessels with little autonomy to fish for a long time and there is little use of vessel tracking systems [78], thus making it virtually impossible to monitor where fishing takes place. In the case of the industrial and semi-industrial fisheries, the boats have an autonomy of roughly three months at sea, and the use of satellite boats is common, with the large one operating as storage for the catch [30].

As a result of this complex scenario, several elasmobranch species commonly bycaught by the gillnet fisheries previously mentioned such as *Sphyrna tudes*, *S*. *tiburo*, *I*. *oxyrhynchus*, *Pristis pristis* and *P*. *pectinata* are now considered to be critically endangered in Brazil [10]. These species have the common feature of neonate and juvenile specimens being consistently found in the BNC’s coastal and estuarine waters [4,84–86], which has been highlighted in previous studies as a potential communal nursery for elasmobranchs [87]. In fact, most of the BNC has been considered as a global conservation hotspot for elasmobranchs due to its high degree of irreplaceability as a crucial habitat for these animals [88].

Recent data using vertebrae microchemistry has demonstrated that the BNC might actually be an essential habitat, especially for species that fulfill their entire life cycle in the area [40]. Indeed, habitat use data indicate that *C*. *porosus* likely uses the waters of the Amazon coast during all life stages, with some level of sexual segregation. This habitat use pattern fits the description provided by Knip et al [89] of how small coastal shark species tend to use their habitat. Therefore, *C*. *porosus* is much more vulnerable to the fishing activity than other migrating coastal species such as *Galeocerdo cuvier* [90]. Despite this, species distribution modelling and historical catch data estimated that the Amazon coast is the area where the species has the highest catch and occurrence probability throughout its distribution range [40]. Therefore, the Amazon coast is likely the most important area for this species throughout its distribution, but is also where fisheries exposure are likely the highest [91].

### Conservation status recommendation

Considering all the data shown and the published information regarding the level of the threats for *C*. *porosus*, including the exposure to different fisheries within the BNC, we applied the IUCN criteria for conservation status assessments [58]. Overexploitation and the elevated juvenile captures, together with an intrinsically low resilience to fisheries, caused a significant decline in *C*. *porosus* population. The demographic analyses in the present study and the exploitation scenarios showed over 90% declines in the population over thirty years ago. Since its generation time is approximately 8 years, at least four generations have passed since the data presented herein was collected. Therefore, *C*. *porosus* fits Criterion E (Quantitative analysis showing the probability of extinction in the wild is at least 50% in three generations) of the IUCN. Even though this study is restricted to the BNC, the species faces similar threats throughout its geographic distribution [92], and thus should be considered as CR globally.
